# The Greater the Number of Altered Eating Behaviors in Obesity, the More Severe the Psychopathology

**DOI:** 10.3390/nu16244378

**Published:** 2024-12-19

**Authors:** Elvira Anna Carbone, Marianna Rania, Ettore D’Onofrio, Daria Quirino, Renato de Filippis, Lavinia Rotella, Matteo Aloi, Vanessa Teresa Fiorentino, Rinki Murphy, Cristina Segura-Garcia

**Affiliations:** 1 Psychiatry Unit, Department of Health Sciences, University “Magna Graecia” of Catanzaro, 88100 Catanzaro, Italy; elvira.carbone@unicz.it (E.A.C.); ettoredo@gmail.com (E.D.);; 2 Outpatient Unit for Clinical Research and Treatment of Eating Disorders, University Hospital Renato Dulbecco, 88100 Catanzaro, Italy; marianna.rania@hotmail.it; 3 Department of Medical and Surgical Sciences, University “Magna Graecia” of Catanzaro, 88100 Catanzaro, Italy; dr.quirinodaria@gmail.com (D.Q.); laviro@hotmail.it (L.R.); vanessa.fiorentino@unicz.it (V.T.F.); 4 Department of Clinical and Experimental Medicine, University of Messina, 98122 Messina, Italy; matteo.aloi@unime.it; 5 Department of Medicine, Faculty of Medical and Health Sciences, University of Auckland, Auckland 1010, New Zealand; murphy@auckland.ac.nz

**Keywords:** obesity, eating behaviors, binge eating, sweet eating, food addiction, night eating, hyperphagia

## Abstract

**Background**: Altered eating behaviors (AEBs) are not only associated with eating disorders but also play a role in obesity. This study assessed AEBs in individuals with obesity and their association with general and eating psychopathology, using the “Eating Behaviors Assessment for Obesity” (EBA-O). The hypothesis posited that a higher frequency of pathological eating behaviors would correlate with more severe psychopathology. **Methods**: Participants seeking weight loss treatment answered the EBA-O and other measures of eating and general psychopathology. The analysis employed MANOVA to estimate psychopathological variance based on AEBs and a cluster analysis to identify patient clusters by AEB type and number. **Results**: Out of the 244 participants, approximately two-thirds reported clinically relevant AEBs, with almost half exhibiting more than two AEBs. Predominant AEBs included sweet eating, binge eating, and hyperphagia. A significant impact of the numbers of AEBs on both eating and general psychopathology severity (*p* < 0.001; η^2^ = 0.167) was evident. Three clusters emerged, with Cluster 3 showing the highest AEB frequency and greater psychopathological impairment. **Conclusions**: The present findings confirm the correlation between the frequency of AEBs and the severity of general and eating psychopathology in individuals with obesity. AEBs deserve clinical attention, and their screening might aid their clinical characterization and foster more tailored treatments.

## 1. Introduction

Obesity is a global pandemic affecting approximately 300 million people worldwide [1]. It is considered one of the ten most risky health conditions, and its medical consequences explain the associated poor quality of life, reduction in life expectancy, and high mortality rates [1,2]. To date, obesity is rated as the fifth leading cause of death globally, with the poorest outcomes being related to the severity of BMI [3]. Obesity is a heterogeneous condition resulting from the interplay of genetic, physiological, behavioral, and environmental factors interacting and contributing to its development, and maintenance [4]. Notwithstanding its clinical relevance, evidence-based treatments designed to promote weight loss still exhibit unsatisfactory results in the long term, and this highlights the urgent need for more effective and personalized approaches [5,6,7].

In this context, efforts have been made to “phenotype” obesity [8,9,10,11] to better understand its complexity and allow healthcare providers to target specific features and risks more effectively. Among these attempts, in the past few years, different altered eating behaviors (AEBs) theorized to contribute to the development and maintenance of obesity have been investigated [12,13]. In particular, food addiction, binge eating, grazing, and night eating have attracted attention in clinical settings and the literature due to their high frequency in high-weight conditions [12,14,15]. Evidence suggests that AEBs are associated with more relevant clinical features, including both general (e.g., emotional distress, depressive and anxiety symptoms) and eating psychopathological aspects (e.g., maladaptive eating patterns, severity of binge eating or night eating symptoms), heightening psychological distress [13,16,17,18,19,20]. Research has also shown the potential of AEBs in phenotyping different clusters of individuals with obesity who might benefit from distinct and targeted treatment approaches [13,20,21,22]. However, many of these AEBs still have no clearly accepted definitions and exhibit overlapping features [15,23,24,25,26,27,28,29,30,31], so their actual incidence remains unclear, as does the chance of studying precision treatments. Hence, the need for validated screening tools to detect and discriminate between AEBs in high-weight conditions is evident [32].

Recently the Eating Behaviors Assessment for Obesity (EBA-O) was developed and validated in a clinical setting [33]. The EBA-O is a self-administered questionnaire and consists of 18 items that account for 5 factors assessing many AEBs (i.e., “food addiction”, “night eating”, “binge eating”, “sweet eating”, and “hyperphagia”). It is an easy-to-use questionnaire delving into AEBs frequently present in obesity.

Although the link between pathological eating behaviors and a patient’s psychopathology has been established, no studies have investigated whether the severity of psychopathology is determined by the number of pathological behaviors or their specific types. A more precise identification of such pathological eating behaviors using the EBA-O could enhance our understanding of their role in clinical and psychopathological outcomes in individuals with obesity. Based on this premise, the current study aimed to assess AEBs in individuals with obesity using the EBA-O to examine their type, frequency, and associations, as well as to evaluate their correlation with both general and specific eating-related psychopathology. Our hypothesis is that a greater number of AEBs, regardless of their type, is associated with more severe psychopathological impairment. This underscores the importance of routine assessment and targeted interventions tailored to these behavioral features.

## 2. Materials and Methods

### 2.1. Participants

Individuals consecutively admitted to the diagnostic and therapeutic network of care for obesity (PDTA Obesità) of the University Hospital “Renato Dulbecco” of Catanzaro (Italy) from January 2022 to June 2024 were screened for eligibility and invited to participate in this study. This network refers to an outpatient program that includes multidisciplinary specialized visits (i.e., internal medicine, surgery, and psychiatry) specifically designed for the assessment and management of people with obesity.

The inclusion criteria were as follows: age between 18 and 65 years, body mass index (BMI) ≥ 30 kg/m^2^, capability to answer self-report questionnaires, and provision of valid informed consent. The presence of severe medical conditions, major psychiatric diagnoses (i.e., neurodevelopmental, schizophrenia spectrum, bipolar disorder, substance-related, and neurocognitive disorders) or pregnancy and breastfeeding over the previous 12 months were considered exclusion criteria. Out of the 246 eligible patients invited, 224 participated and completed the data collection, resulting in a 91% response rate. The main reasons for not participating were lack of interest, limited time, or incomplete questionnaire responses. All participants were informed about the aims, procedures, anonymity, and safety of the data collection before a written informed consent was signed. The study protocol, according to the ethical principles set out in the revised version of the Helsinki Declaration [34], was approved by the Ethical Committee of “Regione Calabria, Sezione Area Centro” (identifier: 162/22 April 2021).

### 2.2. Procedures and Assessment

Participants first underwent a medical visit to evaluate their physical health, nutritional status, and anthropometric data (height, weight, and BMI) and a clinical interview to explore their previous and current eating history, including meal patterns, overeating episodes, food preferences, and emotional triggers for eating. Participants were also asked about their weight history, including onset and progression of obesity, patterns of obesity development at different life stages (i.e., early obesity, late obesity, and adolescent obesity, corresponding to ages 2–5, 6–12, and 13–18 years, respectively), and any previous weight loss attempts. Socio-demographics were also collected. The interview was a combination of open-ended and close-ended questions to ensure comprehensive data collection while allowing participants to provide detailed responses. After the interview, during the same visit, patients answered the following scales and questionnaires:

1. EBA-O [33]: This is a self-administered questionnaire and consists of 18 items that evaluate 5 AEBs (i.e., “food addiction”, “night eating”, “binge eating”, “sweet eating”, and “hyperphagia”) over the previous three months. A cut off score ≥ 4 was applied for the factors and total score to indicate clinically relevant AEBs.
2. Eating Disorder Examination Questionnaire (EDE-Q 6.0) [35]: This is a self-administered questionnaire assessing eating psychopathology over the past 28 days with 36 items, rated 0–6. The tool provides a mean Global Score and four subscales, namely restraint (R), eating concern (EC), shape concern (SC), and weight concern (WC), with higher scores indicating more impaired eating behaviors.
3. State–Trait Anxiety Inventory (STAI) [36]: this is a self-administered questionnaire made up of 40 items that assess state (STAI-S) and trait (STAI-T) anxiety.
4. Beck Depression Inventory (BDI-II) [37]: this is a self-report questionnaire used to assess the presence and severity of depression through 21 items; scores of <10, 10–16, 17–29, and >30, respectively, indicate minimal, mild, moderate, and severe depression.

The average time needed to answer these questionnaires was about 20–30 min.

### 2.3. Data Analysis

Descriptive statistics were computed for socio-demographic and clinical variables as well as for the scores obtained from the psychometric assessment. Data are presented as means and standard deviations (SDs) or as frequencies and percentages (%), appropriately. Chi-square tests were executed to assess the association between the number of current AEBs and sex or BMI category. A Multivariate Analysis of Variance (MANOVA) was carried out with the number of current AEBs from the EBA-O as an independent variable and the total scores from the EDE-Q subscales, BDI-II, and STAI-S as dependent variables. The effect size for the MANOVA was measured using eta-squared (η^2^), where values of 0.01, 0.06, and 0.14 were interpreted as small, medium, and large effects, respectively.

A two-step cluster analysis was performed based on the EBA-O factors (i.e., binge eating, food addiction, sweet eating, night eating, hyperphagia, total number). This method enabled us to handle categorical variables and to automatically determine the optimal number of clusters. It further allows for the identification of groupings through quick cluster algorithms (pre-clustering) and runs hierarchical cluster models in the second step. The log-likelihood criterion was utilized for the distance measure, and Schwarz’s Bayesian criterion (BIC) and the silhouette coefficient were used to compare cluster solutions. A silhouette value under 0.2 was considered poor, between 0.2 and 0.5 was considered fair, and above 0.5 was considered good quality for the solution, with fair or higher considered acceptable clustering [38,39]. Differences between clusters were assessed through Chi-square or MANOVA, as appropriate. Cramer’s V (V) was used to measure the effect size for Chi-square, with values of <0.2, 0.2–0.6, and >0.6 indicating weak, moderate, and strong associations, respectively.

The level of statistical significance was set at a nominal value of *p* ≤ 0.05. Statistical analyses were carried out using the Statistical Package for Social Sciences, Version 26 (SPSS, Chicago, IL, USA).

## 3. Results

The final sample consisted of 224 patients (N = 182 female, 81%; N = 42 male, 19%), with a mean age of 39.4 ± 13.5 years old and a mean BMI of 41.6 ± 7.1 kg/m^2^ (30.3–69.5 kg/m^2^). No effect of sex was evident on age (F = 1.714; *p* = 0.192), BMI (F = 1.280; *p* = 0.259), employment (χ^2^ = 6.236, *df* = 5, *p* = 0.284), education (χ^2^ = 7.664, *df* = 4, *p* = 0.105), and civil status (χ^2^ = 0.958, *df* = 4, *p* = 0.916). Table 1 shows the main characteristics of the participants, and Table 2 summarizes the average scores from the psychometric questionnaires.

**Figure 1:**
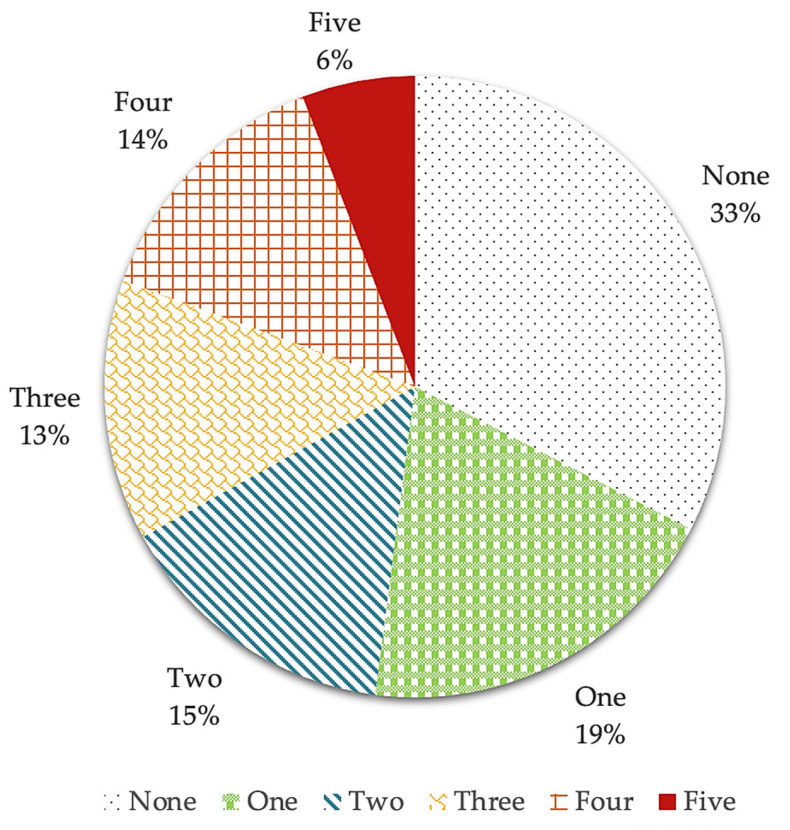
Distribution of the number of AEBs in the sample. The figure displays the percentage of participants with pathological eating behaviors according to the EBA-O.

Nearly 67% of the patients had at least one clinically relevant altered eating behavior according to the EBA-O (mean score ≥ 4) (Figure 1). The frequency of participants with one, two, three, four, or five pathological eating behaviors was, respectively, 19%, 15% 13%, 14%, and 6%. No effect of the BMI (χ^2^ = 15.033, *df* = 10, *p* = 0.131) or sex (χ^2^ = 7.094, *df* = 5, *p* = 0.214) category on the number of AEBs was evident.

**Figure 2:**
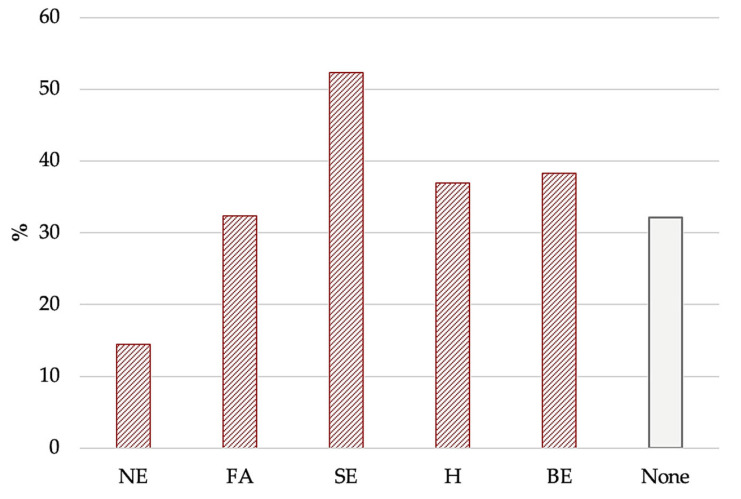
Frequencies of AEBs according to EBA-O. Frequencies of altered eating behaviors according to EBA-O (cut-off ≥ 4). Sweet eating (52%) and night eating (14%) were the most and least frequently reported AEBs, respectively. The column in light grey represents the percentage of patients without altered eating behaviors (EBA-O scores < 4). Abbreviations: BE: *binge eating*; FA: *food addiction*; H: *hyperphagia*; NE: *night eating*; SE: *sweet eating*.

The most frequent and least frequent AEBs were, respectively, sweet eating (52.3%) and night eating (14.5%) (Figure 2); the remaining factors—food addiction (32.4%), hyperphagia, (36.9%), and binge eating (38.3%)—were almost equally distributed. Among the participants reporting AEBs, the most frequent associations were the following:

- Sweet eating + hyperphagia + binge eating + food addiction (14.1%);
- Sweet eating + hyperphagia + binge eating + food addiction + night eating (8.7%);
- Sweet eating + hyperphagia (8.1%);
- Sweet eating + hyperphagia + binge eating (5.4%).

**Figure 3:**
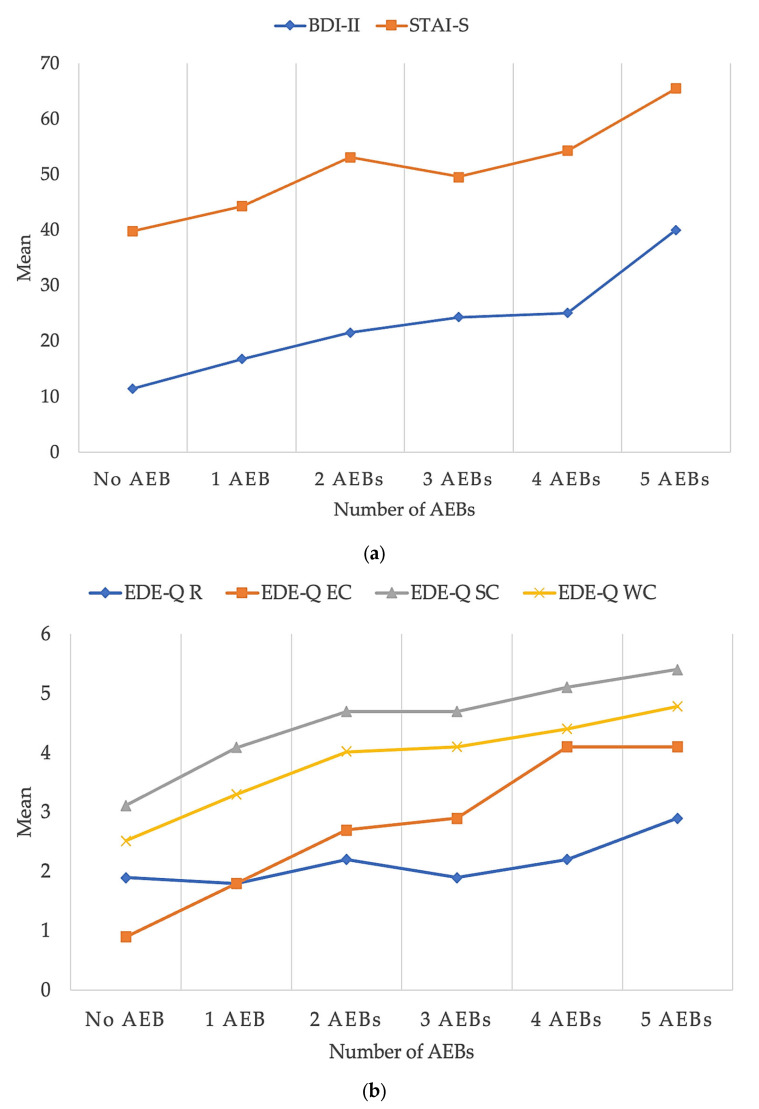
Severity of affective (**a**) and eating (**b**) psychopathology with an increasing number of AEBs. Abbreviations: BDI-II: *Beck Depression Inventory*; EDE-Q: *Eating Disorder Examination Questionnaire*; EDE-Q EC: *eating concern*; EDE-Q R: *restraint*; EDE-Q SC: *shape concern*; EDE-Q WC: *weight concern*; STAI-S: *State Anxiety Inventory*.

A significant effect of the number of current AEBs on the severity of psychopathology was evident (Wilk’s Lambda = 0.401; F = 5.181; *p* < 0.001; η^2^ = 0.167), except for restraint (EDE-Q) (Table 3 and Figure 3).

The two-step cluster analysis, based on the number and types of AEBs, identified three clusters: Cluster 1 comprised 33% of patients (n = 73), Cluster 2 included 35% of patients (n = 78), and Cluster 3 comprised 33% of patients (n = 73). This three-cluster solution gave rise to the highest value for the distance measure (3.004) and the lowest BIC value of −357.081. The silhouette coefficient was 0.6. The main predictors in decreasing order of importance were total number of altered eating behaviors (1.0), binge eating (0.40), food addiction (0.35), sweet eating (0.30), hyperphagia (0.22), and night eating (0.12).

Table 4 depicts the cluster comparison. Cluster 3 reported a significantly higher number of AEBs compared to the other clusters (Cluster 1 = 0 ± 0; Cluster 2 = 1.5 ± 0.6; Cluster 3 = 3.8 ± 0.8; F = 930.999; *p* < 0.001; η^2^ = 0.894). Significantly lower rates of late childhood obesity (χ^2^ = 11.579, *df* = 2, *p* = 0.003, V = 0.237) or adolescent obesity (χ^2^ = 12.468, *df* = 2, *p* = 0.002, V = 0.245) were evident in Cluster 1 compared to Clusters 2 and 3. No significant differences emerged for age (F = 1.230, *p* = 0.294), sex (χ^2^ = 0.050, *df* = 2, *p* = 0.975), and BMI (F = 2.288, *p* = 0.104).

**Figure 4:**
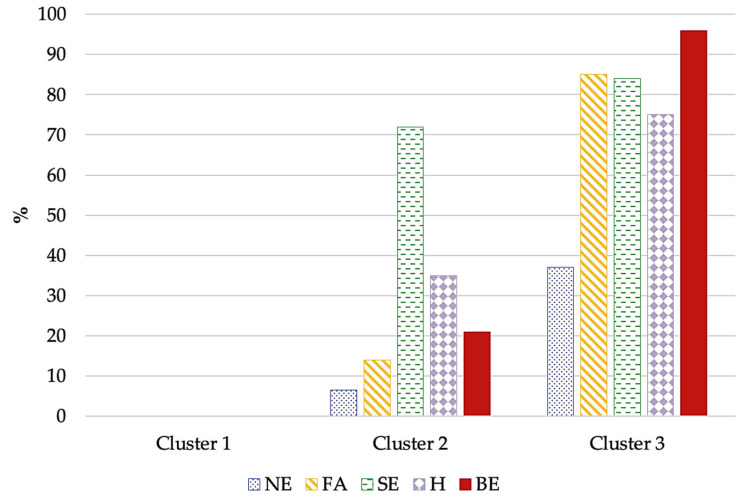
AEB distribution in the three clusters. Cluster 1: no altered eating behavior (AEB); Cluster 2: a few AEBs—with prevalent sweet eating; Cluster 3: many AEBs. Abbreviations: BE: *binge eating*; FA: *food addiction*; H: *hyperphagia*; NE: *night eating*; SE: *sweet eating*.

Significant differences in the distribution AEBs were evident: patients in Cluster 1 reported no AEBs; Cluster 2 was characterized by participants with sweet eating behavior; meanwhile, Cluster 3 was symptomatically depicted by the presence of all AEBs (Figure 4).

**Figure 5:**
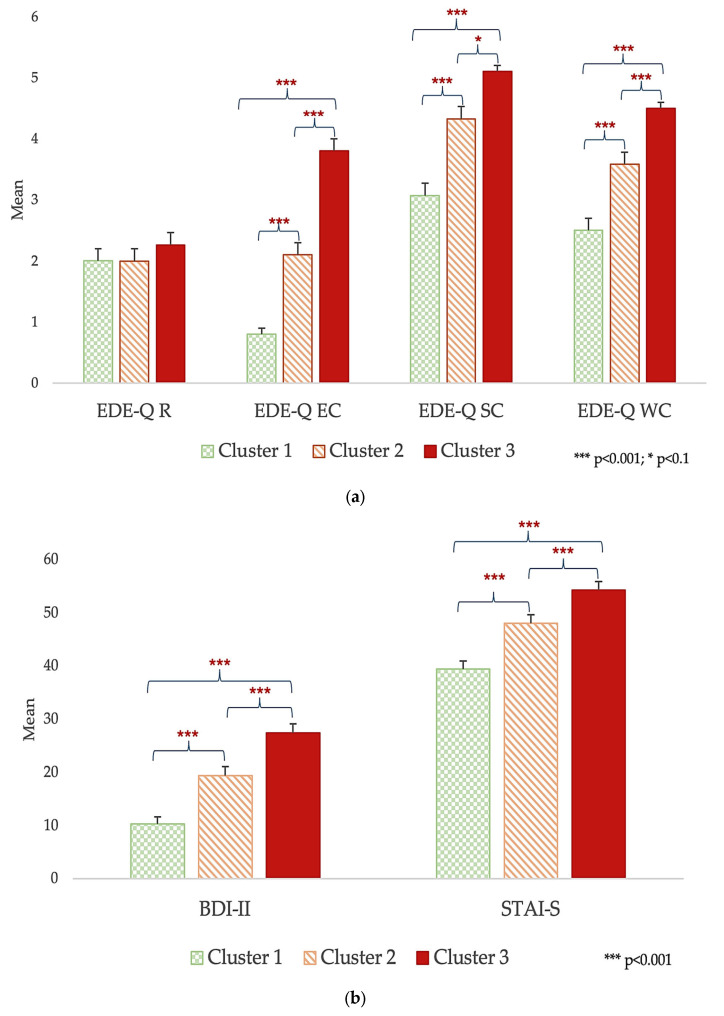
Cluster comparison on psychopathological variables. (**a**) Eating psychopathology: patients in Cluster 3 exhibited higher scores on all EDE-Q subscales, except for restraint, compared to those in other clusters. (**b**) Affective symptoms: patients in Cluster 3 scored higher on the STAI-S and BDI-II than those in other clusters. Abbreviations: BDI-II: *Beck Depression Inventory*; EDE-Q: *Eating Disorder Examination Questionnaire*; EDE-Q EC: *eating concern*; EDE-Q R: *restraint*; EDE-Q SC: *shape concern*; EDE-Q WC: *weight concern*; STAI-S: *State Anxiety Inventory*. Data are expressed as means and standard errors of the mean.

The MANOVA also revealed other significant differences between clusters (F = 9.879, *p* < 0.001, η^2^ = 0.275): Cluster 3 had significantly higher scores in the EDE-Q EC (F = 62.621, *p* < 0.001, η^2^ = 0.438), EDE-Q SC (F = 23.942, *p* < 0.001, η^2^ = 0.229), EDE-Q WC (F = 33.539, *p* < 0.001, η^2^ = 0.294), STAI-S (F = 16.104, *p* < 0.001, η^2^ = 0.167), and BDI-II (F = 24.944, *p* < 0.001, η^2^ = 0.237) (Figure 5). No significant differences were evident in the EDE-Q R (F = 0.822, *p* = 0.441).

## 4. Discussion

Research has recently paid attention to AEBs, especially binge eating, grazing, and food addiction, aiming to investigate their associations with obesity [27,28,31,40]. This study investigated the type, frequency, and associations of AEBs and their correlation with general and eating psychopathology in a clinical sample of individuals seeking weight loss treatment for obesity.

To date, this is the first comprehensive examination of AEBs in a clinical sample of individuals with obesity [27,28,40,41,42,43]. Remarkably, two-thirds of the participants reported at least one clinically relevant AEB, and half of them exhibited two or more. The present results further demonstrated a positive association between the cumulative number of AEBs as well as the type of AEB and the clinical severity of general and eating psychopathology.

Evidence on this topic has demonstrated that when obesity and AEBs overlap, the clinical features and eating psychopathology are more relevant [13,15,19,27,30,31,44,45] and mental health-related quality of life decreases [27,31,45]. The presence of different eating behavior patterns in patients suffering from obesity might hinder several explanations. Altered self-perceptions and the sensory/hedonic evaluation of foods due to dysregulated serotoninergic, dopaminergic, and opioidergic brain pathways might influence eating behaviors [21,46]. According to the escape theory [47,48], patients may eat as a way of coping with negative emotions or because they may confuse physiological changes related to emotions (i.e., stress, depression, and sadness) with internal states of hunger and satiety [49,50]. Even when satiety is achieved, the availability of highly palatable food may overrule satiety and increase meal sizes, contributing to an increased intake of food and, ultimately, to obesity [51]. Finally, underlying metabolic conditions (e.g., reactive hypoglycemia) may also be associated with some AEBs in obesity [52].

The results of the cluster analysis further confirmed that the number, as well as the type, of AEBs affects both general and eating-specific psychopathology. Based on the number and types of AEBs, three clusters emerged: Cluster 1: “No AEBs”; Cluster 2: “Few AEBs”; and Cluster 3: “Many AEBs”, respectively, associated with absent/mild, moderate, and severe clinical symptomatology. On the other hand, considering the type of AEBs, Cluster 1 could be labeled as “simple obesity”; Cluster 2, with only prevalent sweet eating, could be labeled as “sweet-tooth obesity”; and Cluster 3, showing all AEBs, especially binge eating and food addiction, could be labeled as “complex obesity”. Again, the frequency of AEBs in the clusters appeared to be associated with the severity of psychopathology. Cluster 3 had significantly higher scores in eating, shape, and weight concern, as well as higher anxiety and depressive symptoms. So, in our sample, the more disordered eating behaviors there are, the worse the psychopathology. AEBs such as binging or food addiction are associated with body image disperception, low self-esteem, depression, anxiety, increased perceived life stress, and lower quality of life [53,54]. People suffering from obesity are often more dissatisfied with their bodies than normal-weight individuals [55]: as BMI increases, the evaluation of appearance worsens, weight and shape concern rises [56], and body dissatisfaction is augmented. This, in turn, may exasperate low self-esteem or depressive symptoms and reinforce and perpetuate the AEBs. The present results do support differences in weight and shape concern according to the number and types of AEBs (i.e., belonging to a definite cluster) but not according to BMI.

The present data support and confirm previous findings: patients suffering from obesity are not all the same, and AEBs may help clinicians in identifying different phenotypes of obesity [20]. It may be hypothesized that Cluster 3 could contain a higher proportion of patients who are at risk of an eating disorder (ED), such a binge eating disorder (BED), than Clusters 1 and 2. Studies have demonstrated that AEBs can progress to an ED [57,58] and that binge eating, food addiction, or sweet eating are often reported among patients with EDs, particularly in BED [22,42,59]. Thus, it is important to detect signs of disordered eating habits early and to treat them right away.

When present, AEBs may influence the outcome of treatments that are not multitargeted. There is evidence suggesting that AEBs in patients with obesity are associated with a higher BMI at present and across their lifetime, restrictive diets, and cyclic weight changes [28,43,60]; poorer weight loss treatment outcomes [27,31,45] are also reported after bariatric surgery, worsening outcomes in terms of quality of life [16]. In this study, Clusters 2 and 3 self-reported higher rates of late childhood or adolescent obesity. It has already been shown that a higher BMI in childhood is a risk factor not only for disordered eating [61] but also for eating disorders (e.g., BED, bulimia nervosa) [62], leading to higher levels of eating and general psychopathology severity and comorbid mental health difficulties [62]. AEBs, even without the diagnosis of an eating disorder, may have serious health consequences (e.g., obesity, diabetes, metabolic syndrome) [13,63]. The adequate characterization of patients seeking weight loss treatment according to the types and number of AEBs seems, at this point, essential to selecting the most effective and personalized treatment. In light of this, an integrated approach (i.e., nutritional, medical, psychological, and pharmacological) would be preferred to treat comorbid AEBs in patients suffering from obesity.

Before reaching a conclusion, the authors recognize that this study has some potential limitations. First, the cross-sectional design does not allow for understanding the direction of the relationship between the development of the AEBs and the observed psychopathology. Second, self-report questionnaires, although well-validated tools and consistent with previous research, may be affected by the recall bias, leading to the under/overestimation of both AEBs and affective symptoms. Third, men were underrepresented in the sample, potentially hindering the detection of different prevalences of AEBs or related implications for psychopathology. Thus, further studies with a larger sample and a longitudinal design could provide more robust evidence and generalizable findings. Likewise, exploring the different environmental and biological aspects of eating behaviors more deeply and further investigation for addressing the challenging issue of obesity are important.

On the other hand, this study has some strengths, such as the comprehensive examination of AEBs in a real-world clinical sample of individuals with obesity seeking weight loss treatment. It offers insights into the association between AEBs and eating psychopathology, with a large sample size and the use of well-validated and easy-to-use psychometric tools. The cluster analysis, additionally, shows the diversity of AEBs among individuals with obesity, supporting the assumption that the number and types of AEBs have a critical bearing in psychopathology.

## 5. Conclusions

The present findings confirmed the hypothesis that a higher number of AEBs is associated with greater general psychopathological (e.g., anxiety and depression) and eating psychopathological severity in individuals with obesity. AEBs require attention and treatment as they may turn into more problematic eating disorders and heighten the risk of serious health complications. Eating behaviors significantly impact patients’ health, especially with regard to weight control. Early interventions for AEBs may help individuals to achieve healthier eating habits and manage their weight more effectively, thus reducing the risk of obesity-related health conditions (e.g., diabetes or cardiovascular disorders). Additionally, from the potential health risks associated with AEBs derives the need for comprehensive treatment strategies that can encompass a wide variety of approaches, including nutritional counseling, psychological therapy, and medication support tailored to the specific needs of the individual patient. Finally, by addressing AEBs as quickly as possible, patients may have a chance of avoiding the development of more severe and potentially debilitating conditions like EDs.

## Figures and Tables

**Table 1:** Sample description.

		Fr	%
Education	Elementary school	9	4
	Middle school I	55	25
	High school II	107	48
	University degree	40	18
	No answer	13	6
Employment	Unpaid activity	32	14
	Employed	88	39
	Unemployed	48	21
	Student	36	16
	On pension	6	3
	No answer	14	6
Civil status	Single	74	33
	Married	123	55
	Divorced	12	5
	Widowers	1	1
	No Answer	14	6
Early childhood obesity	Yes	57	25
Late childhood obesity	Yes	107	48
Adolescent obesity	Yes	141	63
BMI category	I (30–34.99 kg/m^2^)	34	15
	II (35–39.99 kg/m^2^)	76	34
	III (≥40 kg/m^2^)	114	51

**Table 2:** Descriptive statistics from the psychometric assessment.

		Mean	SD	Min	Max
EDE-Q	R	2.0	1.5	0	6
	EC	2.2	1.7	0	6
	SC	4.1	1.6	0	6
	WC	3.5	1.5	0	6
	Total	3.0	1.3	0	5.8
BDI-II	Total	19.2	13.7	0	54
STAI-S	Total	47.5	14.2	20	80
STAI-T	Total	49.1	14.9	20	79
EBA-O	NE	1.5	1.9	0	7
	FA	2.7	2.1	0	7
	SE	3.9	2.3	0	7
	H	2.8	2.2	0	7
	BE	2.7	2.4	0	7
	Total	2.7	1.7	0.05	7

Abbreviations: BDI-II: Beck Depression Inventory; BE: binge eating; EBA-O: Eating Behaviors Assessment for Obesity; EDE-Q EC: eating concern; EDE-Q R: restraint; EDE-Q SC: shape concern; EDE-Q WC: weight concern; FA: food addiction; H: hyperphagia; NE: night eating; SE: sweet eating; STAI-S and T: State–Trait Anxiety Inventory.

**Table 3:** MANOVA: between-subject effects.

Dependent Variable	F	*p*	η^2^
BDI-II	14.039	**<0.001**	0.310
STAI-S	10.194	**<0.001**	0.246
EDE-Q R	1.529	0.184	0.047
EDE-Q EC	30.967	**<0.001**	0.498
EDE-Q SC	9.860	**<0.001**	0.240
EDE-Q WC	14.746	**<0.001**	0.321

(Independent variable: number of current altered eating behaviors). Abbreviations: η^2^ = eta square; BDI-II: *Beck Depression Inventory*; EDE-Q: *Eating Disorder Examination Questionnaire*; EDE-Q EC: *eating concern*; EDE-Q R: *restraint*; EDE-Q SC: *shape concern*; EDE-Q WC: *weight concern*; STAI-S: *State Anxiety Inventory*. Significant results are in bold.

**Table 4:** Cluster comparison.

		Cluster 1 N = 73		Cluster 2 N = 78		Cluster 3 N = 73				
		Mean	SD	Mean	SD	Mean	SD	Statistics	*p*	Effect Size
Number of AEBs		0	0	1.5	0.6	3.8	0.8	F = 930.999	**<0.001**	η^2^ = 0.894
Age		40.5	12.7	40.4	13.7	37.3	14.1	F = 1.230	0.294	
BMI		42.8	8.4	41.7	6.1	40.3	6.6	F = 2.288	0.104	
Sex ^§^	Female	59	81	64	82	59	81	χ^2^ = 0.050	0.975	
	Male	12	19	14	18	14	19			
Early childhood obesity ^§^		14	21	26	36	17	26	χ^2^ = 3.960	0.138	
Late childhood obesity ^§^		24	36	47	64	36	54	χ^2^ = 11.579	**0.003**	V = 0.237
Adolescent obesity ^§^		36	53	58	81	47	70	χ^2^ = 12.468	**0.002**	V = 0.245

^§^ data are expressed as frequencies and percentages. Effect size only displayed for significant results. η^2^ = eta square; V = Cramer’s V. Significant results are in bold.

## Data Availability

Data supporting the findings of this study are available from the corresponding author upon reasonable request. The data are not publicly available because patients were not asked for permission, and they did not give consent to share their information publicly.
